# ANOCA, INOCA, MINOCA: The New Frontier of Coronary Syndromes

**DOI:** 10.3390/jcdd12020064

**Published:** 2025-02-10

**Authors:** Antonio L. M. Parlati, Ermanno Nardi, Vincenzo Sucato, Cristina Madaudo, Giulio Leo, Tanisha Rajah, Federica Marzano, Maria Prastaro, Paola Gargiulo, Stefania Paolillo, Giuseppe Vadalà, Alfredo Ruggero Galassi, Pasquale Perrone Filardi

**Affiliations:** 1 Department of Advanced Biomedical Sciences, University of Naples Federico II, 80131 Naples, Italy; 2 Division of Cardiology, Department of Excellence of Sciences for Health Promotion and Maternal-Child Care, Internal Medicine and Specialties (ProMISE) “G. D’Alessandro”, Paolo Giaccone Hospital, University of Palermo, 90133 Palermo, Italy; 3 Cardiology Division, Department of Biomedical, Metabolic and Neural Sciences, University of Modena and Reggio Emilia, Policlinico di Modena, 41121 Modena, Italy; 4 Birmingham Medical School, University of Birmingham, Birmingham B15 2TT, UK

**Keywords:** INOCA, ANOCA, MINOCA, IHD

## Abstract

The growing prevalence in the diagnosis of INOCA (Ischemia with Non-Obstructive Coronary Arteries), ANOCA (Angina with Non-Obstructive Coronary Arteries), and MINOCA (Myocardial Infarction with Non-Obstructive Coronary Arteries) highlights the need to reassess their clinical relevance. Historically regarded as benign syndromes, emerging evidence suggests that these conditions may cause serious cardiovascular events and considerable long-term disability. Additionally, emerging studies suggest that non-obstructive coronary artery disease (CAD) may have a higher prevalence compared to traditional obstructive forms of CAD. This leads to the need to better clarify the underlying pathogenic mechanisms as well as the risk factors associated with these syndromes. This is precisely the aim of this review, which focuses on the complex and heterogeneous mechanisms underlying these syndromes as well as the associated risk factors. This review also sums up the diagnostic steps necessary to achieve an accurate diagnosis, along with the interventional and pharmacological approaches to be implemented in light of the latest evidence.

## 1. Introduction

The prevalence of ischemic heart disease (IHD) is constantly increasing, representing a major cause of morbidity and mortality worldwide. It is estimated that over 250 million people are affected by IHD, with a high incidence in low- and middle-income countries [1]. IHD is the leading cause of death globally, contributing to approximately 9.4 million deaths in 2021 and responsible for approximately 1 in 7 deaths [2]. The epidemiological data about this pathology are expected to increase constantly by 2050 [2]. In recent years, the panorama of IHD has broadened its diagnostic and therapeutic horizons, focusing on a group of coronary syndromes that do not fit into the traditional paradigms of epicardial coronary artery obstruction. Among these, ANOCA (Angina with Non-Obstructive Coronary Arteries), INOCA (Ischemia with Non-Obstructive Coronary Arteries) and MINOCA (Myocardial Infarction with Non-Obstructive Coronary Arteries) represent complex and often underdiagnosed conditions characterized by the presence of anginal symptoms and/or evidence of myocardial ischemia in the absence of detectable obstructive coronary lesions in traditional coronary angiography [3]. These conditions frequently stem from microvascular dysfunction, coronary vasospasms, or abnormalities in blood flow regulation that require a sophisticated assessment for a precise diagnosis and treatment. This narrative review will explore in detail the definition, clinical characteristics, pathophysiology, and most recent diagnostic and therapeutic strategies for ANOCA/INOCA and MINOCA. The objective is to provide an in-depth and updated overview, with particular attention to the most relevant scientific advances and to the challenges still open in the management of these conditions, which are redefining the contemporary approach to IHD in clinical practice.

## 2. Angina with Non-Obstructive Coronary Arteries (ANOCA) and Ischemia with Non-Obstructive Coronary Arteries (INOCA)

### 2.1. Definition

ANOCA is a syndrome characterized by the presence of anginal symptoms or ischemic equivalent symptoms in patients who do not present with significant stenosis of the epicardial coronary arteries on a coronary angiographic examination, including functional evaluation of intermediate stenoses with techniques such as fractional flow reserve (FFR) and instantaneous wave-free ratio (iFR) [4]. INOCA is a condition characterized by the evidence of myocardial ischemia documented by non-invasive functional testing without the presence of significant stenoses in the epicardial coronary arteries, evidenced by coronary angiography. While the ANOCA definition focuses on the symptom, the INOCA definition focuses on the evidence of myocardial ischemia. As of this moment, considering the common pathophysiological mechanisms, diagnostic processes, and therapeutic strategies of these conditions, we will refer to them with the joint term ANOCA/INOCA.

### 2.2. Epidemiology

ANOCA/INOCA represent common but underestimated clinical conditions. Recent studies estimate that 40–70% of patients undergoing elective coronary angiography for angina symptoms have ANOCA, and 20–30% of these patients have INOCA [4,5,6,7,8]. These conditions are common in individuals with cardiovascular (CV) risk factors [6]. A portion of 50–70% of ANOCA/INOCA patients are women, and 10–20% of women who underwent coronary revascularization had a previous ANOCA/INOCA [9,10]. These conditions are particularly prevalent in post-menopausal women, where hormonal changes could contribute to increased vulnerability to endothelial dysfunction, coronary vasospasm, and microvascular dysfunction [11].

### 2.3. Pathophysiology

The pathophysiology is multifactorial and involves different functional and structural mechanisms that mainly affect the coronary microcirculation and the endothelium. The epicardial coronary arteries primarily perform a conductance function, providing a path for coronary blood flow (CBF) to the myocardium. Under physiological conditions, these arteries do not directly regulate flow in response to the heart’s oxygen demand [12]. The coronary microcirculation, instead, is crucial for adapting CBF to the metabolic needs of the myocardium. This compartment is composed of arterioles and capillaries that can change their tone to increase or decrease CBF in response to changes in oxygen demand [13]. The increased demand for oxygen during stressful conditions induces, in physiological conditions, a vasodilatory response affecting both the epicardial arteries and the microcirculation through the release of biochemical mediators, such as adenosine, nitric oxide (NO), and prostacyclins, by the endothelium leading to an increase in the coronary flow reserve (CFR) [12,13]. ANOCA/INOCA patients frequently have coronary microvascular dysfunction (CMD) or vasospastic angina (VSA) in the absence of significant epicardial stenosis with a mismatch in blood supply and myocardial oxygen demands, which consequently causes angina and/or myocardial ischemia [14,15]. Among patients with ANOCA/INOCA, approximately two-thirds show signs of CMD [4,6]. Recently, CMD has been classified as structural (non-endothelium-dependent) or functional (endothelium-dependent) [4]. The structural form results from an arteriolar remodeling process, common to numerous other structural heart diseases associated with the coexistence of CV risk factors such as smoking, hypertension, dyslipidemia, and diabetes [15,16,17,18,19,20]. The structural changes affecting the arterioles include an increase in parietal thickness with a rarefaction of the microcirculation due to the proliferation of the parietal smooth muscle cells, perivascular fibrosis, and thickening of the intimate tunic [12,13]. These structural changes can lead to an increased index of microvascular resistance (IMR) and an impaired CFR [4]. The functional form, instead, derives from an inappropriate arteriolar vasodilatory response, which is mediated by endothelial dysfunction. In the case of endothelial dysfunction, the physiological vasodilatory response is reduced or even replaced by vasoconstriction. Vascular smooth muscle cells can also exhibit functional alterations and contribute directly to pathogenesis [12]. Finally, epicardial coronary vasospasm results from inappropriate vasoconstriction of vessel smooth muscle cells in response to exposure to a wide range of stimuli, including smoking, medications, stress, hyperventilation, and cold exposure [9]. Vagal hypertonicity also appears to be predisposing, explaining the circadian pattern of incidence [21]. Furthermore, previous studies have supported an association with obstructive and non-obstructive atherosclerosis [22,23]. In particular, a study conducted by Shin and colleagues highlighted a significant prevalence of eroded plaques, often associated with the presence of a thrombus in segments with documented spasms [23]. Depending on whether the duration is transient or prolonged, the spasm can result in VSA or myocardial infarction [9].

### 2.4. Diagnosis

Patients with ANOCA/INOCA typically experience angina, which is clinically indistinguishable from an epicardial obstructive coronary disease. In addition to angina, other symptoms, including dyspnea, weakness, nausea, and/or irregular sleep patterns, can be present [4,24]. It is important to identify the presence of CV risk factors such as hypertension, diabetes, and dyslipidemia and perform an evaluation of cardiac markers, lipid profiles, glycemia, and inflammatory markers [4,25]. A resting electrocardiogram (ECG) and exercise test can also be important tools to detect ECG abnormalities suggestive of ischemia, but these tests are often normal in patients with ANOCA/INOCA [9]. Many non-invasive tools for the evaluation of inducible ischemia, such as stress echocardiography, single photon emission tomography (SPECT), positron emission tomography (PET), and cardiac magnetic resonance (CMR), have a limited role in the diagnosis of ANOCA/INOCA. Indeed, regional anomalies of the movement or the perfusion of the ventricular walls are rarely found in these patients, probably due to a diffuse and “patchy” distribution of the ischemia, which is not limited to a single territory of vascularization [26,27]. However, these tools can play a role in the endothelium-independent CMD. Indeed, the endothelium-independent CMD can also be investigated using non-invasive methods like the measurement of coronary flow reserve (CFR) after intravenous administration of endothelium-independent vasodilators such as adenosine, regadenoson, or dipyridamole [9]. For example, transthoracic echocardiography (ETT) allows the measurement of, via pulsed spectral Doppler, the flow in basal and hyperemic conditions at the level of the mid-distal segment of the anterior descending artery, showing excellent reproducibility and concordance with the CFR measurement carried out in an invasive manner [28]. Likewise, PET and CMR allow the measurement of coronary/myocardial flow reserve through the use of radioactive tracers and gadolinium, respectively [9,29]. The recent 2024 European Society of Cardiology (ESC) guidelines about the management of chronic coronary syndromes recommended the use of the non-invasive methods mentioned above with a IIb class of evidence in the evaluation of CMD due to their intrinsic limitation of not being able to exclude epicardial coronary obstruction, suggesting a sequential testing with an anatomical imaging like coronary computed tomography angiography (CCTA) to suspect the diagnosis of ANOCA/INOCA [4]. However, especially in the endothelium-dependent CMD, a definitive diagnosis can only be obtained with invasive evaluation. After the evaluation of the epicardial coronary vessels, including the functional evaluation of epicardial intermediate stenoses with FFR/iFR, a combination of techniques to evaluate the microcirculation and the vasomotor function of the coronary tree through specific diagnostic guides and the administration of pharmacological agents must be performed through a standardized protocol, generally at the level of the left anterior descending coronary artery [4,9]. CFR represents the most used parameter to define CMD. It is given by the ratio between CBF in hyperemia and at rest and represents the ability of the coronary tree to increase myocardial blood flow in response to an increase in oxygen demand; a CFR value of < 2.5 is considered pathological [30,31]. However, considering its compartmental non-specificity, the CFR (obtained using adenosine with dedicated catheters equipped with Doppler sensors [30] or based on the principle of thermodilution [29,30]) becomes a relatively accurate index of the microcirculation functionality exclusively in the absence of functionally significant epicardial coronary disease. More specific indices of microvascular functionality are the IMR (obtained through the use of thermodilution) and the hyperemic microvascular resistance index (HMR, obtained through the Doppler technique) [32]. IMR values of >25 U or HMR >2.5 mmHg/cm/s define the presence of endothelium-independent CMD [3]. A combined assessment of CFR and IMR/HMR allows a better characterization of the pathophysiological mechanisms underlying CMD: structural (CFR < 2.5 and IMR > 25/HMR > 2.5) or functional (CFR < 2.5 and IMR < 25/HMR < 2.5) [4]. Instead, patients with VSA predominantly present with angina at rest, and their diagnosis is mainly based on a provocative vasomotility test performed in the cath laboratory, the “gold standard” for the diagnosis of this subtype of INOCA/ANOCA [4,33,34]. Although numerous vasoactive substances have been tested [35], acetylcholine (Ach) is the pharmacological agent of first choice [4]. It is an agonist of the parasympathetic nervous system, and its cholinergic muscarinic receptors are located at the level of the endothelium and smooth muscle fibrocells. Ach promotes endothelium-mediated vasodilation in healthy subjects and elicits vasoconstriction in coronary arteries susceptible to spasms or in the presence of endothelial dysfunction [36]. The provocative test is performed during coronary angiography by administering intracoronary Ach at increasing doses (up to a maximum of 200 μg in the standardized protocol proposed in the ESC guidelines [4]) with a continuous monitoring 12-lead ECG and angiography image acquisition after each drug injection to detect the appearance of vasospasm, anginal symptoms or ECG changes [36,37,38]. The interpretation of this test is based on the diagnostic criteria drawn up by the International Group for the Study of Coronary Vasomotility Disorders (COVADIS). An epicardial spasm [34] is defined as epicardial vasoconstriction of > 90% on coronary angiography associated with the reproduction of anginal symptoms and ischemic alterations on ECG, while a microvascular spasm is outlined in the case of the coexistence of anginal symptoms and ischemic changes on the ECG in the absence of significant epicardial angiographic alterations [36]. The diffusion in clinical practice has been hindered by doubts regarding the safety of this test. However, recent evidence revealed that adverse events during provocative tests are infrequent and transitory, encouraging their use [36,39]. Invasive and non-invasive techniques for the diagnosis of ANOCA/INOCA are detailed in Table 1.

### 2.5. Treatment

The treatment of ANOCA/INOCA is based on individualized management, which considers the underlying cause and specific risk factors [3,7]. The CorMicA trial investigated the impact of an interventional diagnostic procedure (IDP) paired with personalized medical therapy on the health outcomes of 151 patients with angina and no obstructive disease. The IDP involved guidewire-based measurements of coronary flow reserve, microcirculatory resistance index, and fractional flow reserve, followed by an acetylcholine vasoreactivity test. The study demonstrated that this approach is both practical for routine clinical use and effective in alleviating angina symptoms in patients with non-obstructive CAD [40].

**Figure 1:**
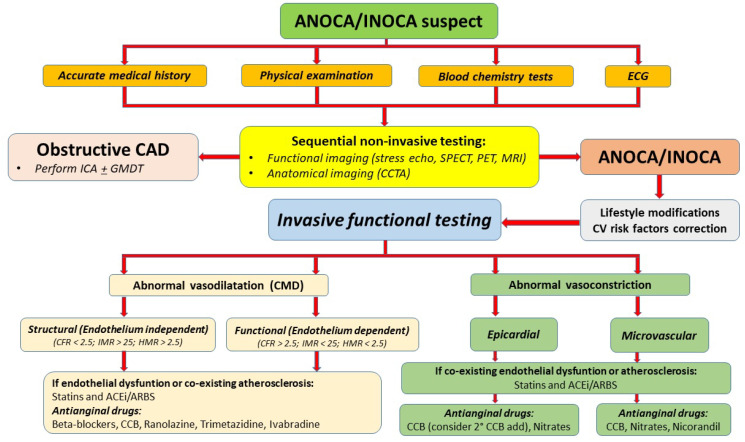
Management of ANOCA/INOCA. ACEi: angiotensin-converting enzyme inhibitors; ANOCA: Angina with Non-Obstructive Coronary Arteries; ARBs: angiotensin II receptor blockers; CAD: coronary artery disease; CCB: calcium channel blocker; CCTA: coronary computed tomography angiography; CMD: microvascular dysfunction; CFR: coronary flow reserve; GMDT: guideline-directed medical therapy; CV: cardiovascular; ECG: electrocardiogram; HMR: hyperemic microvascular resistance index; ICA: invasive coronary angiography; IMR: index of microvascular resistance; INOCA: ischemia with non-obstructive coronary arteries; MRI: cardiac magnetic resonance; PET: positron emission tomography; SPECT: single photon emission tomography.

The three cornerstones of ANOCA/INOCA therapy are represented by lifestyle changes, the control of CV risk factors, and pharmacological therapy [41]. Strict control of CV risk factors through statins, antihypertensive and antidiabetic drugs, weight loss, and smoking cessation is recommended, according to the most recent ESC guidelines [4]. In particular, angiotensin-converting enzyme inhibitors (ACE inhibitors), angiotensin II receptor blockers (ARBs), calcium channel blockers (CCB), and statins have protective effects on endothelial function and the prevention of epicardial spasm [42,43,44]. Traditional first-line antianginal therapies for ANOCA/INOCA are beta-blockers and CCB [4]. In particular, carvedilol and nebivolol have antioxidant properties and promote vasodilation through the release of NO, improving vascular resistance and CFR [45,46,47]. In the case of a lack of or an inadequate response, the combination of beta-blockers with dihydropyridine CCB and/or the addition of long-acting nitrates can be considered [4]. Second-line therapies include ranolazine (a sodium channel blocker that reduces intracellular calcium levels with consequently muscle relaxation, increased myocardial perfusion, and improvement of microvascular function) and trimetazidine (reduces ischemia by promoting the use of glucose through inhibition of fatty acid metabolism) [48,49]. Some patients with persistent symptoms could even benefit from treatment with ivabradine, which reduces the heart rate by inhibiting the If current of the sinoatrial node without any inotropic effect. However, the results of its effectiveness are still controversial as some studies have not shown significant improvements in angina symptoms or quality of life compared to conventional therapies. For example, Villano and colleagues observed that ivabradine has no effects on coronary microvascular function and on flow-mediated dilation compared to a placebo [50]. In approximately 25% of patients, the symptoms remain refractory to the aforementioned therapeutic strategies. In epicardial and microvascular coronary vasospasm, instead, first-line treatment consists of non-dihydropyridine CCB and/or short-acting nitrates [4]. In this context, sometimes the dosage of verapamil and diltiazem exceeds the commonly recommended posology, and it is imperative to avoid the use of non-selective beta-blockers [4,51]. For the epicardial coronary vasospasm, a second-line therapy includes nicorandil, a drug promoting the relaxation of smooth muscle cells and the synthesis of NO, with both a vasodilatory and beneficial effect on the microcirculation [4,52]. Nowadays, for the treatment of ANOCA/INOCA, promising therapies are still being studied, including endothelin receptor antagonists (such as zibotentan, inhibiting the action of endothelin, a potent vasoconstrictor [53]), Rho kinase inhibitors (such as fasudil, reducing the contractility of the vascular wall [54]) and xanthine derivatives (such as aminophylline, reducing the release of adenosine during exercise [55]). Finally, the role of implantation of a coronary sinus reduction device in the treatment of microvascular dysfunction is yet to be demonstrated [56]. The management of ANOCA/INOCA is detailed in Figure 1.

### 2.6. Prognosis

The prognosis of ANOCA/INOCA is variable and depends on the severity of coronary dysfunction and the timeliness and effectiveness of the treatment [4]. Patients with ANOCA/INOCA have a lower mortality risk than patients with epicardial obstructive coronary heart disease but higher than the general population, especially in patients with severe CMD [57,58]. Many patients have a stable course of pathology, but some patients may experience recurrent angina and functional limitations significantly impairing their quality of life [57]. Other patients can also develop evident coronary atherosclerosis over time [9]. Furthermore, patients with INOCA are at increased risk of developing CV events such as non-fatal myocardial infarction and heart failure compared to patients with angiographically normal epicardial vessels, with an annual adverse event rate of 2–5% [9,59].

## 3. Myocardial Infarction with Non-Obstructive Coronary Arteries (MINOCA)

### 3.1. Definition

Myocardial Infarction with Non-Obstructive Coronary Arteries (MINOCA) is a form of acute myocardial infarction (AMI) in which angiography reveals no obstructive lesions in the epicardial coronary vessels. Over time, various definitions of MINOCA have emerged. In 2017, Agewell et al., in a position paper by the European Society of Cardiology (ESC), outlined the criteria necessary to define MINOCA [60];

AMI is defined by the current universal definition of epicardial coronary arteries with angiographic lesions not exceeding 50% of the vessel diameter.

An absence of other clinically evident specific causes that could explain the clinical presentation.

**Figure 2:**
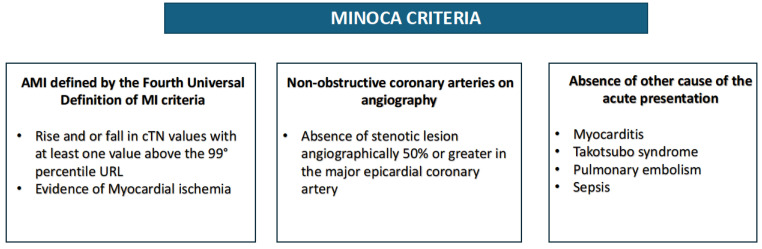
Diagnostic criteria of MINOCA. MINOCA: Myocardial Infarction with Non-Obstructive Coronary Arteries.

In 2019, Tamis-Holland et al., in a scientific statement from the American Heart Association (AHA), reiterated the concept, specifying that MINOCA, following the “Fourth Universal Definition of MI”, can only be diagnosed in the presence of a rise and/or fall in troponin levels (cTn) with at least one value above the 99th percentile upper reference limit, along with concurrent clinical, electrocardiographic, and/or morphological evidence of myocardial ischemia [61,62] (Figure 2).

### 3.2. Epidemiology

Prevalence data regarding MINOCA are challenging to determine due to evolving diagnostic criteria over time and the complexity of the diagnostic process, which may not be applied in the same way across all countries. However, a 2015 meta-analysis reported an overall prevalence of suspected MINOCA of 6% among more than 176,000 patients diagnosed with AMI [63]. A more recent meta-analysis from 2021, conducted on over 800,000 AMI patients, reported an overall prevalence of suspected MINOCA of 8.1% [64]. An interesting finding appears to be the higher prevalence among younger patients, as observed in a recent multicenter study conducted on patients aged 18 to 55, where a prevalence of suspected MINOCA of 11% was reported [65]. MINOCA patients tend to be predominantly female [63]. Additionally, compared to obstructive MI patients, MINOCA patients have a lower prevalence of hyperlipidemia but similar prevalences of other cardiovascular risk factors [63]. A pooled analysis revealed that around one-third of MINOCA patients presented with myocardial infarction with ST-segment elevation (STEMI), while the other two-thirds were classified as having AMI without ST-segment elevation (NSTEMI) [63].

### 3.3. Pathophysiology

**Figure 3:**
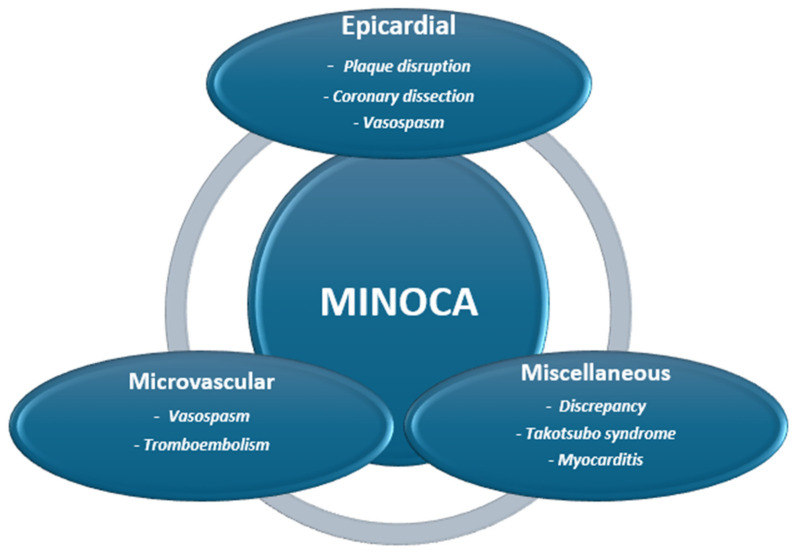
Pathogenetic mechanism of MINOCA. MINOCA: Myocardial Infarction with Non-Obstructive Coronary Arteries.

MINOCA is a complex condition with various potential causes that can be identified using specific algorithms, including intravascular imaging modalities, such as optical coherence tomography (OCT), intravascular ultrasound (IVUS), and cardiovascular magnetic resonance (CMR). Indeed, as highlighted in the ESC position paper, MINOCA should be seen as a working diagnosis, serving as the initial step toward determining the underlying cause [60]. According to the underlying pathogenetic mechanism, MINOCA can be divided into three categories (Figure 3):

Epicardial causes:

- Plaque disruption;
- Coronary dissection;
- Epicardial Vasospasm.

Microvascular causes:

- Microvascular vasospasm;
- Thromboembolism.

Miscellaneous causes (discrepancy, Takotsubo syndrome, myocarditis, other).

#### 3.3.1. Plaque Disruption

Three different types of plaque disruption can contribute to MINOCA: plaque rupture, plaque erosion, and calcified nodules [66]. These disruptions are often undetectable with coronary angiography, making intravascular imaging essential for their identification. IVUS studies have shown that plaque disruption occurs in roughly one-third of MINOCA cases [67,68]. OCT, a high-resolution imaging method, allows for precise assessment of coronary tissue structures, distinguishing between thin cap fibroatheromas and differentiating plaque rupture from erosion [69,70]. Plaque disruption triggers a thrombotic process that can lead either to peripheral embolization and/or vasospasm or transient occlusion of the coronary followed by spontaneous lysis [66,71]. Regardless of the consequences of the thrombotic process, to suggest MINOCA, the degree of stenosis detectable on coronary angiography must always be less than 50% [72].

#### 3.3.2. Coronary Dissection

Spontaneous coronary artery dissection (SCAD)-related MI is a well-recognized cause of MI in women under 50 [73]. SCAD often leads to the formation of an intramural hematoma, which can compress the coronary artery lumen and impair blood flow, leading to MINOCA [74]. Given that SCAD is a common cause of MI in younger women, intracoronary imaging, which can detect SCAD, should be considered for diagnosing MINOCA in this specific group [75].

#### 3.3.3. Coronary Vasospasm

Coronary vasospasm is a common cause of MINOCA arising from an excessive response of the smooth muscle of the coronary wall to endogenous or exogenous vasoconstrictor substances. As a result, important vasoconstriction can occur in the epicardial district, resulting in epicardial vasospasm, or in the microcirculation, resulting in microvascular vasospasm, ultimately impairing myocardial perfusion [61]. In both cases, MINOCA can occur. As established by the ESC guidelines, a definitive diagnosis of vasospasm requires clinical-angiographic documentation, which involves performing intracoronary provocative tests with ACh or ergonovine (ER) [76]. Following COVADIS criteria, the test is deemed positive for epicardial vasospasm if, during its administration, anginal symptoms occur simultaneously along with electrocardiographic changes and coronary spasm, resulting in a luminal reduction of more than 90% compared to the reference caliber of the vessel [34]. According to COVADIS criteria, the diagnosis of microvascular vasospasm requires the onset of anginal symptoms and electrocardiographic changes in the absence of severe epicardial vasoconstriction during an invasive provocative test with ACh or ER [38]. In a recent study by Montone et al., 80 patients with MINOCA underwent provocative testing for vasospasm within 48 h of admission, yielding a positive result in 46% of cases, with approximately two-thirds in the epicardial vessels and one-third in the microcirculation [37]. The same study also noted a significantly worse prognosis for patients with a positive test in terms of more frequent occurrences of cardiac and all-cause mortality, as well as recurrent MI [37].

#### 3.3.4. Thromboembolism

Coronary thromboembolism (CE) is a well-known cause of MINOCA, although it can also be associated with obstructive MI. CE can be divided into direct, paradoxical, and iatrogenic forms [77]. Direct forms occur when the embolic source originates in the left chambers of the heart or from endocarditic processes affecting the mitral or aortic valves. These direct forms are the most common, with atrial fibrillation accounting for roughly 73% of cases [78]. Paradoxical embolisms involve the formation of a thrombus in the venous system and the following passage via an intracardiac shunt, such as foramen ovale or an extracardiac one, as a pulmonary arteriovenous malformation into coronary arteries. The iatrogenic form refers to embolization in coronary arteries of air or devices such as coronary stents following percutaneous coronary interventions (PCIs) or aortic valve replacements [79]. Given the lack of consistent epidemiological data regarding cases of MINOCA caused by CE, it is essential to rule out all conditions that could contribute to thromboembolism, such as atrial fibrillation and endocarditis, as well as hereditary or acquired thrombophilic conditions, such as factor V Leiden mutation or myeloproliferative disorders, that may play a role in CE events.

#### 3.3.5. Miscellaneous Causes

Several miscellaneous disorders fall under the definition of MINOCA, including conditions that create an acute imbalance between oxygen supply and demand in the absence of coronary obstruction, such as anemia, brady/tachyarrhythmias, or hypo/hypertension [62]. This category also includes Takotsubo syndrome (TTS) and myocarditis, although these conditions have specific and distinct characteristics and, therefore, according to many authors, should not be considered causes of MINOCA.

### 3.4. Diagnosis

**Figure 4:**
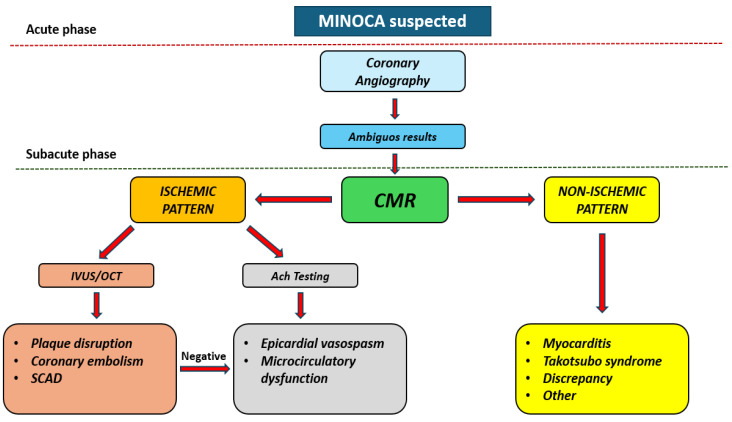
Diagnostic algorithm of MINOCA. CMR: cardiac magnetic resonance; IVUS: intravascular ultrasound; MINOCA: Myocardial Infarction with Non-Obstructive Coronary Arteries; OCT: optical coherence tomography.

The various underlying causes of MINOCA, as well as the wide range of patients who may be affected, require a dynamic approach when managing suspected cases [60]. Although it is not always possible to identify the cause that triggered MINOCA, as clarified by the recent ESC guidelines, it is useful to adopt a specific algorithm made up of multiple steps in order to define the underlying cause [76] (Figure 4).

The first step requires the diagnostic criteria for MI to be met, and other non-ischemic, cardiac, or non-cardiac conditions must be excluded [61]. Coronary angiography is, of course, the primary examination to be performed, with timing according to ECG alterations.

The second step in the diagnostic algorithm is represented by non-invasive imaging, with the extensive use of CMR, which, on the one hand, confirms the presence or absence of an infarcted area and, on the other, excludes alternative diagnoses such as myocarditis and TTS, enabling a definitive diagnosis in over 85% of cases [80,81]. However, about 8–67% of MINOCA cases present with normal MRI results and no evidence of ventricular motion abnormalities, edema, or late gadolinium enhancement (LGE) [82].

An additional complementary step is the use of invasive coronary imaging and coronary physiology tests, useful for plaque characterization and the diagnosis of SCAD, as well as for the diagnosis of coronary spasm and microcirculatory dysfunction [83]. Notably, if suspicion of MINOCA is high, invasive intracoronary tests can reasonably be performed during the index coronary angiography in order to avoid subjecting the patient to two invasive procedures [76].

Some specific etiological suspicions require a particular personalized diagnostic pathway. For instance, in the case of suspected coronary embolism, it is useful to perform a Holter ECG to detect subclinical atrial fibrillation and, if necessary, transesophageal echocardiography for a thorough search for potential embolic sources [61].

Recent studies have concentrated on identifying and evaluating potential biomarkers to assist in determining the cause of and enhancing prognostic stratification in MINOCA. Ciliberti et al. have shown that individuals with MINOCA may have a pro-inflammatory predisposition, as evidenced by elevated biomarkers such as white blood cells and C-reactive protein [84]. It has also been observed that hyperglycemia may occur during the acute phase of MINOCA [85]. Indeed, inflammation and hyperglycemia-mediated alterations can contribute to increased atherosclerotic burden and plaque destabilization or cause vasoconstriction of the coronary arteries and/or promote vasospasm, leading to myocardial ischemia. Additionally, hyperglycemia and elevated inflammatory markers during the acute phase of MINOCA have been associated with a worse prognosis both in the short and long term [86]. In a pooled analysis, it was found that 14% of MINOCA patients undergoing specific screening had an overall prevalence of thrombophilic disorders [63].

### 3.5. Treatment

The absence of randomized clinical trials leads to a lack of clear and consensus-based guidelines for the treatment of MINOCA. Similar to obstructive MI, a low dose of aspirin is recommended for secondary prevention after suspected MINOCA, although the evidence supporting its long-term benefit is still debating [61,87]. On the contrary, dual antiplatelet therapy (DAPT) has not been shown to significantly reduce the major adverse cardiovascular events (MACE) and, therefore, not recommended at all [87,88]. A post-hoc analysis of the randomized CURRENT-OASIS 7 trial, which compared high-dose and standard-dose clopidogrel, even indicated a possible unfavorable prognostic effect of high-dose clopidogrel in MINOCA patients compared to those with obstructive MI [89]. Furthermore, routine use of DAPT should also be avoided in cases of SCAD, as it is associated with a significant increase in major cardiovascular complications, as shown by the analysis of the Italian-Spanish DISCO registry on coronary dissections [90]. It is plausible that DAPT may play a biological role only in cases of “true” ischemic MINOCA with proven evidence of complicated coronary plaque [89].

In some observational studies, statin treatment has been shown to effectively reduce all-cause death and CV events in patients with suspected MINOCA, while in other reports, this benefit was not observed [88,90,91].

Similarly, controversial results have been demonstrated for angiotensin-converting enzyme inhibitors (ACE inhibitors) and Sartans, as well as for beta-blockers [88,92,93]. According to the potential role of inflammation in MINOCA, the use of anti-inflammatory medications like colchicine seems promising, although there is currently no direct evidence of benefit [83,94].

When vasospasm is clearly identified as the etiological mechanism, calcium channel blockers have proven to be the most effective drugs, in combination with or as an alternative to nitrates [61,77].

For patients with MINOCA caused by microcirculatory dysfunction, dipyridamole, and ranolazine are recommended due to their vasodilatory effects, although the main evidence derives from the chronic rather than the acute setting [61].

Cardiac rehabilitation through physical exercise is crucial in managing cardiovascular diseases, as it helps lower mortality rates and the risk of adverse cardiovascular events.

### 3.6. Prognosis

Despite initially being considered a benign event compared to MI, over time, the data have become conflicting [95]. In recent years, several research groups have assessed the real prognostic impact of MINOCA [2]. The predictive factors of mortality are similar to traditional ones, particularly the ECG presentation with ST-segment elevation and the clinical onset of heart failure (HF) or cardiogenic shock [1,61,63,96,97]. In the SWEDEHEART registry, the incidence of all-cause death, reinfarction, stroke, or hospitalization for HF was 13.4%, 7.1%, 4.3%, and 6.4%, respectively, over a mean follow-up of 4.1 years [93]. More recently, in a large meta-analysis conducted on over 55,000 patients with suspected MINOCA, 12 months after the event, an unadjusted reinfarction rate of 2.6%, a major CV event prevalence of nearly 10%, and an all-cause death rate of 3.4% were observed, which was lower than in patients with obstructive MI (5.6%, *p* < 0.001) but far from negligible [64]. Other high-risk subgroups appear to include patients with evidence of diffuse coronary atheroma involving three vessels or the left main coronary artery, as well as those with elevated C-reactive protein levels at admission [84,98]. Moreover, the quality of life, including exercise capacity, also appears to be compromised in patients with MINOCA [99]. Finally, recent autopsy studies have shown that MINOCA can lead to sudden death, frequently affecting young individuals, with 43% of cases involving a history of stimulant substance or drug abuse, with significant social and preventive implications [100,101].

## 4. Conclusions

The insights from this review call for a significant change in the approach of ANOCA, INOCA, and MINOCA. These conditions are complex and require further investigation into their clinical impacts and the mechanisms behind them. It is important to note that the severity of ischemia in these patients does not always match the level of atherosclerosis in their coronary arteries or the intensity of their angina. Furthermore, it is worth emphasizing that these patients are not at low risk; if left without an appropriate diagnosis and/or treatment, they can face a higher chance of cardiovascular events. In light of this, a multidisciplinary approach to patients with non-obstructive coronary arteries is required. It is crucial to have a more personalized approach to the diagnosis and treatment of these conditions that considers the risk factors associated with these syndromes as well as the clinical signs and underlying mechanisms involved.

## Figures and Tables

**Table 1:** Invasive and non-invasive techniques for the diagnosis of ANOCA/INOCA. Ach: acetylcholine; ANOCA: Angina with Non-Obstructive Coronary Arteries; CFR: coronary flow reserve; CMR: cardiac magnetic resonance; FFR: fractional flow reserve; HMR: hyperaemic myocardial resistance; iFR: instantaneous wave-free ratio; IMR: index of microvascular resistance; INOCA: Ischemia with Non-Obstructive Coronary Arteries; PET: positron emission tomography; VSA: vasospastic angina.

Non-Invasive Techniques				
Test	Advantages	Disadvantages	Challenges	Feasibility
**Stress Echocardiography**	- No radiation - Low cost - Ischemia and CFR evaluation	- Limited for ANOCA/INOCA - Dependent on operator skills	- Limited in patients with poor acoustic windows (echo contrast use)	- Available in advanced cardiac centers (mostly hospital centers)
**PET**	- Flow and perfusion quantification - CFR evaluation	- Radiation exposure - High cost	- Specific radiotracers needed, limited availability	- Limited access (available in specialized centers)
**CMR**	- High resolution - No radiation exposure - Precise myocardial scar location - Perfusion and CFR evaluation	- High cost - Long duration - Contraindications - Functional analysis limited in arrhythmias - Limited 3D quantification of ischemia	- Experience required for interpretation	- Limited access (available in specialized centers)
**CCTA**	- Non-invasive anatomical evaluation - Non-invasive FFR	- Limited for microvascular function evaluation - Radiation exposure - Image quality limited in arrhythmias	- Experience required for interpretation	- Moderate availability, requires advanced equipment
**Invasive Techniques**				
**Test**	**Advantages**	**Disadvantages**	**Challenges**	**Feasibility**
**FFR/iFR**	- Epicardial intermediate stenosis evaluation	- Invasive - Specific wires required - No microvascular function evaluation	- Requires specific experience	- Limited to centers with advanced catheterization laboratory
**Invasive CFR, IMR/HMR**	- Detailed evaluation of microvascular function	- Invasive - Vasodilator drugs required	- Complex interpretation - Variable protocols	- Limited to centers with advanced catheterization laboratory
**Ach/Ergonovine test**	- Gold standard for VSA	- Risk of complications - Not always diagnostic	- Perceived security - Need for standardized protocols	- Available only in centers with specific expertise
